# Identification of New Degrons in *Streptococcus mutans* Reveals a Novel Strategy for Engineering Targeted, Controllable Proteolysis

**DOI:** 10.3389/fmicb.2017.02572

**Published:** 2017-12-19

**Authors:** Nan Liu, Muhammad T. Chaudhry, Zhoujie Xie, Jens Kreth, Justin Merritt

**Affiliations:** ^1^Department of Restorative Dentistry, School of Dentistry, Oregon Health and Science University, Portland, OR, United States; ^2^Pakistan Council of Scientific and Industrial Research, Islamabad, Pakistan; ^3^MOE Key Laboratory of Industrial Fermentation Microbiology, College of Biotechnology, Tianjin University of Science and Technology, Tianjin, China; ^4^Department of Molecular Microbiology and Immunology, School of Medicine, Oregon Health and Science University, Portland, OR, United States

**Keywords:** protein targeting, proteolysis, ribonuclease, *Streptococcus mutans*, NEDD8, degron, FtsH, ClpXP

## Abstract

Recently, controllable, targeted proteolysis has emerged as one of the most promising new strategies to study essential genes and otherwise toxic mutations. One of the principal limitations preventing the wider adoption of this approach is due to the lack of easily identifiable species-specific degrons that can be used to trigger the degradation of target proteins. Here, we report new advancements in the targeted proteolysis concept by creating the first prokaryotic N-terminal targeted proteolysis system. We demonstrate how proteins from the LexA-like protein superfamily can be exploited as species-specific reservoirs of N- and/or C-degrons, which are easily identifiable due to their proximity to strictly conserved residues found among LexA-like proteins. Using the LexA-like regulator HdiR of *Streptococcus mutans*, we identified two separate N-degrons derived from HdiR that confer highly efficient constitutive proteolysis upon target proteins when added as N-terminal peptide tags. Both degrons mediate degradation via AAA+ family housekeeping proteases with one degron primarily targeting FtsH and the other targeting the ClpP-dependent proteases. To modulate degron activity, our approach incorporates a hybrid N-terminal protein tag consisting of the ubiquitin-like protein NEDD8 fused to an HdiR degron. The NEDD8 fusion inhibits degron function until the NEDD8-specific endopeptidase NEDP1 is heterologously expressed to expose the N-degron. By fusing the NEDD8-degron tag onto GFP, luciferase, and the pleiotropic regulator RNase J2, we demonstrate that the N-terminal proteolysis approach exhibits far superior performance compared to the classic transcriptional depletion approach and is similarly applicable for the study of highly toxic mutations.

## Introduction

The ability to exogenously control protein levels in cells has long been an invaluable tool to study cellular and molecular biology. In bacteria, this is typically achieved via genetic knock-out mutations or through overexpression. However, in many instances it is necessary to engineer an exogenous control mechanism to regulate gene expression for such studies, especially when knock-out mutations or overexpression constructs result in severe toxicity or lethality (Saida et al., 2006). This is most often achieved using induction systems that regulate gene expression at the transcriptional level via the exogenous control of inducible promoters. For toxic mutations, such systems can be employed to create conditional lethal mutations of essential genes via a transcriptional depletion approach. Numerous induction cassettes are widely available for use in many different species and these systems are generally straightforward to engineer in genetically tractable organisms (Bertram and Hillen, 2008; Brautaset et al., 2009; Wang et al., 2012; Singh et al., 2014; Myronovskyi and Luzhetskyy, 2016; Wolf and Mascher, 2016). Consequently, transcriptional depletion is among the most common mechanisms to study the function of essential genes. Despite this, the transcriptional depletion approach is subject to several limitations that can be inherently problematic for studies of gene function. Many proteins exhibit high intrinsic stability and are quite slow to turnover naturally once they have been produced. For such proteins, it can take multiple generations after initiating transcriptional depletion before a measurable phenotypic response occurs (Guzman et al., 1995; Ji et al., 2001; McGinness et al., 2006; Griffith and Grossman, 2008; Davis et al., 2011; Kim et al., 2011; Wei et al., 2011). Slow or inefficient depletions can also be particularly problematic for toxic mutations, since it allows for the development of compensatory gene regulation or suppressor mutations (Davis et al., 2011). Furthermore, the unnatural expression patterns of target genes controlled by induction cassettes can potentially trigger unintended artifacts due to cis and even trans effects upon heterologous gene expression (Wei et al., 2011; Cameron and Collins, 2014; Liu et al., 2015). In addition, many genes are subject to potent posttranscriptional regulatory mechanisms that can effectively override induced changes in transcription (Papenfort and Vogel, 2010; Condon and Bechhofer, 2011; Durand et al., 2015; Papenfort and Vanderpool, 2015; Wagner and Romby, 2015; Dendooven and Luisi, 2017). A variety of more recent approaches have been developed to circumvent these limitations. One approach involves the use of synthetic small-molecule-controlled riboswitches like the theophylline riboswitch, which can be engineered into transcripts to modulate the translational efficiency of target genes (Topp et al., 2010). Currently available riboswitch systems have yet to match the broad range of tunability exhibited by most induction cassettes (Topp et al., 2010; Etzel and Morl, 2017; Folliard et al., 2017). The newest approach is to engineer controllable proteolysis into proteins of interest. Since this approach directly targets specific proteins, it is much less susceptible to the limitations encountered with induction systems.

Thus far, only a handful of controllable proteolysis systems have been developed for use in bacteria and all exploit the SsrA C-degron (C-terminal proteolytic degradation epitope) encoded by the tmRNA (McGinness et al., 2006; Griffith and Grossman, 2008; Davis et al., 2011; Kim et al., 2011; Wei et al., 2011; Cameron and Collins, 2014). The tmRNA is a highly conserved prokaryotic protein quality control system used to rescue stalled ribosomes by terminating their translation and targeting the resulting truncated polypeptides for degradation (Moore and Sauer, 2007; Keiler, 2008; Barends et al., 2011). In most Gram positive and Gram negative bacteria, proteins containing the SsrA C-degron are primarily targeted to the housekeeping ClpXP protease (Gottesman et al., 1998; Wiegert and Schumann, 2001; Lies and Maurizi, 2008; Ahlawat and Morrison, 2009; Tao and Biswas, 2015), but in some species like *Mycoplasma florum* the Lon protease plays a major role (Gur and Sauer, 2008). These proteases all belong to the AAA+ superfamily and hydrolyze ATP to unfold target proteins and then rapidly degrade them processively (Sauer and Baker, 2011; Olivares et al., 2016). Three general strategies have been developed to exploit the function of the SsrA C-degron for targeted proteolysis. The first utilizes the adaptor protein SsrB, which enhances ClpXP proteolytic degradation of SsrA tagged proteins in some species like *Escherichia coli* and *Caulobacter crescentus* (Levchenko et al., 2000; Bolon et al., 2004; Lessner et al., 2007). By mutating the *E. coli* or *C. crescentus* SsrA C-degron, it is possible to weaken its recognition by the ClpXP protease and increase its dependence upon SsrB for efficient degradation (McGinness et al., 2006). Controlled proteolysis can then be achieved by appending the modified *E. coli* or *C. crescentus* degron onto the C-terminus of target proteins, while concurrently placing the corresponding *E. coli* or *C. crescentus ssrB* gene under the control of an induction cassette (McGinness et al., 2006; Griffith and Grossman, 2008; Davis et al., 2011; Kim et al., 2011). The second approach is to engineer a target protein with an endogenous species-specific SsrA C-degron that has been masked by adding an endopeptidase cleavage site immediately after the SsrA tag (Wei et al., 2011). Next, the appropriate endopeptidase is placed under the control of an induction cassette. Since the SsrA C-degron is only functional when exposed at the C-terminus, target proteins will remain stable until the endopeptidase has been expressed. The third approach exploits the unusual specificity of the *M. florum* SsrA C-degron for the Lon protease. Since the *M. florum* SsrA C-degron is not recognized by other species, it is possible to add its SsrA peptide as a C-terminal degron to proteins of interest (Cameron and Collins, 2014). Next, the *M. florum* Lon protease is placed under the control of an induction cassette to modulate the stability of targeted proteins. As each of these three approaches utilize induction cassettes, variable levels of proteolysis can be selected by adjusting the level of induction in the assay. Furthermore, the exceptional efficiency of protein degradation via housekeeping proteases like ClpXP makes it feasible to rapidly and thoroughly deplete the cell of highly abundant, stable proteins.

In the current study, we describe new advancements in the controllable proteolysis concept. We have developed the first N-terminal proteolysis system in bacteria by combining novel SsrA-independent degrons with a new, high-specificity endopeptidase cleavage system to regulate targeted proteolysis. Our approach exploits the untapped reservoir of endogenous degrons embedded within LexA orthologs and other LexA-like proteins. Since LexA-like proteins are found in most, if not all bacteria, our approach should be easily adaptable for use in a wide range of species.

## Experimental procedures

### Bacterial strains, plasmids, and culture conditions

Bacterial strains, plasmids, and their relevant characteristics are listed in Table 1, while the primers used for strain and plasmid construction are listed in Table 2. All *Streptococcus mutans* strains were grown anaerobically (85% N_2_, 10% CO_2_ and 5% H_2_) at 37°C in Todd-Hewitt medium (Difco) with 0.3% yeast extract (THYE medium). For the selection of antibiotic-resistant transformants, THYE plates were supplemented with 800 μg ml^−1^ kanamycin (Sigma), 900 μg ml^−1^ spectinomycin (Sigma), or 12 μg ml^−1^ erythromycin (Sigma).

**Table 1:** Strains and plasmids.

**Strains and plasmids**	Relevant characteristics^*^	**References**
**STRAINS**		
UA159	Wild-type *S. mutans*	
GN10P	UA159 Δ*irvR*, Δ*clpP*, pGN10, Km^r^, Em^r^, Sp^r^	Niu et al., 2010
WTLH	UA159::*ldh-HA*, markerless	Liu et al., 2015
S1-ftsH	UA159::*gyrA_*P*_*::*xylR, ldh_*P*_*::*xylA_*O*_-ftsH*, Km^r^	Unpublished
Cflip-gfp	WTLH::*ldh_*p*_-C-flip-gfp*, Sp^r^	This work
Ndeg-gfp	WTLH::*ldh_*p*_-N-degron-gfp*, Sp^r^	This work
irvRN-gfp	WTLH::*ldh_*p*_-irvR N-tag-gfp*, Sp^r^	This work
NdegP-gfp	WTLH::*ldh_*p*_-N-degron-gfp, ΔclpP*, Sp^r^, Em^r^	This work
CflipP-gfp	WTLH::*ldh_*p*_-C-flip-gfp, ΔclpP*, Sp^r^, Em^r^,	This work
CflipB-gfp	WTLH::*ldh_*p*_-C-flip-gfp, ΔclpB*, Sp^r^, Km^r^	This work
NdegB-gfp	WTLH::*ldh_*p*_-N-degron-gfp, ΔclpB*, Sp^r^, Km^r^	This work
CflipH-gfp	WTLH::*ldh_*p*_-C-flip-gfp, gyrA_*P*_*::*xylR, ldh_*P*_*::*xylA_*O*_-ftsH*, Sp^r^, Km^r^	This work
NdegH-gfp	WTLH::*ldh_*p*_-N-degron-gfp, gyrA_*P*_*::*xylR, ldh_*P*_*::*xylA_*O*_-ftsH*, Sp^r^, Km^r^	This work
smSENP1	WTLH + pSENP1, Sp^r^	This work
smNEDP1	WTLH + pNEDP1, Sp^r^	This work
smUlp1	WTLH + pUlp1, Sp^r^	This work
Cflip-bdS	WTLH::*ldh_*p*_-bdSUMO+C-flip-gfp*, pSENP1, Km^r^, Sp^r^	This work
Cflip-bdN	WTLH::*ldh_*p*_-bdNEDD8+C-flip-gfp*, pNEDP1, Km^r^, Sp^r^	This work
Cflip-scS	WTLH::*ldhp-scSUMO+C-flip-gfp*, pUlp1, Km^r^, Sp^r^	This work
Ndeg-bdS	WTLH::*ldh_*p*_-bdSUMO+N-degron-gfp*, pSENP1, Km^r^, Sp^r^	This work
Ndeg-bdN	WTLH::*ldh_*p*_-bdNEDD8+N-degron-gfp*, pNEDP1, Km^r^, Sp^r^	This work
Ndeg-scS	WTLH::*ldh_*p*_-scSUMO+N-degron-gfp*, pUlp1, Km^r^, Sp^r^	This work
BDJ2	WTLH::*ldh_*p*_-bdNEDD8+C-flip-rnjB*, pNEDP1, Km^r^, Sp^r^	This work
XYLJ2	WTLH::*gyrA_*P*_*::*xylR, ldh_*P*_*::*xylA_*O*_-rnjB*, Sp^r^	This work
iRenGIV	UA159::*ldh_*P*_*-*lucR*; *gyrA_*P*_*::*xylR, ldh_*P*_*::*xylA_*O*_-renG*; Kan^R^	Merritt et al., 2016
Cflip-renG	UA159::*ldh_*p*_-bdNEDD8+C-flip-renG*, pNEDP1, Km^r^, Sp^r^	This work
Ndeg-renG	UA159::*ldh_*p*_-bdNEDD8+N-degron-renG*, pNEDP1, Km^r^, Sp^r^	This work
XYLrenG	UA159::*gyrA_*P*_*::*xylR, ldh_*P*_*::*xylA_*O*_-renG*, Sp^r^	This work
Cflip-gfp2	WTLH::*ldh_*p*_-hdirC2-gfp* (C-flip 4 amino acid tag), Sp^r^	This work
Cflip-gfp3	WTLH::*ldh_*p*_-hdirC3-gfp* (C-flip 6 amino acid tag), Sp^r^	This work
Cflip-gfp4	WTLH::*ldh_*p*_-hdirC4-gfp* (C-flip 15 amino acid tag), Sp^r^	This work
Ndeg-gfp2	WTLH::*ldh_*p*_-hdirN2-gfp* (N-degron 4 amino acid tag), Sp^r^	This work
Ndeg-gfp3	WTLH::*ldh_*p*_-hdirN3-gfp* (N-degron 6 amino acid tag), Sp^r^	This work
Ndeg-gfp4	WTLH::*ldh_*p*_-hdirN4-gfp* (N-degron 15 amino acid tag), Sp^r^	This work
**PLASMIDS**		
pDL278	*E. coli-Streptococcus* shuttle vector, Sp^r^	Chen and Leblanc, 1992
pWVKTs	Temperature sensitive shuttle vector, Km^r^	Gutierrez et al., 1996
pZX9	pVA380::*gyrA_*P*_*::*xylR, ldh_*P*_*::*xylA_*O*_*::*luc*, Sp^r^	Xie et al., 2013
pSENP1	pDL278::*gyrA_*P*_*::*xylR, ldh_*P*_*::*xylA_*O*_-bdSENP1*, Sp^r^	This work
pNEDP1	pDL278::*gyrA_*P*_*::*xylR, ldh_*P*_*::*xylA_*O*_-bdNEDP1*, Sp^r^	This work
pUlp1	pDL278::*gyrA_*P*_*::*xylR, ldh_*P*_*::*xylA_*O*_-scUlp1*, Sp^r^	This work

* *Sp^r^, spectinomycin resistance; Em^r^, erythromycin resistance; Km^r^, kanamycin resistance*.

**Table 2:** Primers.

**Primer**	Sequence (5′-3′)^*^	**Relevant constructs**
HdirC-gfp F1	**ATGGCATCATTAAAAAGAATACGTTAGATACCCCTAT**ATGTCAAAAGGAGAAGAGCTGTTC	Cflip-gfp
ldhp RC1	**ACGTATTCTTTTTAATGATGCCAT**GTATCTAACGTATTCTTTTTAATGATGCCAT	
HdirN-gfp F1	**ATGGGCACAGGCTATTCCTATTTTGGTGATGGCAAT**ATGTCAAAAGGAGAAGAGCTGTTC	Ndeg-gfp
ldhp RN1	**TAGGAATAGCCTGTGCCCAT**GTTCTAAACATCTCCTTATAATTTATTA	
irvRN-gfp F1	**ATGGGTACTGGTATCTGGGTTGGACATGAAAAA**ATGTCAAAAGGAGAAGAGCTGTTC	*S. mutans* irvRN-gfp
ldhp RRN1	**AACCCAGATACCAGTACCCAT**GTTCTAAACATCTCCTTATAATTTATTA	
ldhp F	**CTAATTGGTAAGCGCGCCATG**GTAAATATTAGTGACTTTCTTAACAA	irvRN-gfp, Cflip-gfp, and Ndeg-gfp
gfp R	TTACTTATAAAGCTCATCCATGCCGTGAG	
ldh F	**ATGGATGAGCTTTATAAGTAA**TTATAAGGAGATGTTTAGAACAT	
ldh R	TTAAGCGTAATCTGGAACATCGTATGGGTAGTTACGAGCTGCAGCAGCAAATT	
ldh up F	CTTCAATGCGTTTTTCTTGTGCTAAGCGAAC	
ldh up R	**TTATAACATGTATTCACGAACGAAAATC**AGCTGAAAAAGAGCCTTATT TGTGATATA	
ClpP UP F	CATTTCGCATCCGCTCATTCAGCACAAACTCT	CflipP-gfp and NdegP-gfp
ClpP DN R	ATTCAAGATTGTAACCAATTTTTACAGTATCTCC	
ClpB UP F	GTGTCAACAGGCTCAGACCTTGAA	CflipB-gfp and NdegB-gfp
ClpB UP R	**CGGTATAATCTTACCTATCACCT**AAATCTCATTTTATCGATCATAAAGTCACCTC	
ClpB DN F	**TCTAAAAGTTCGCTAGATAGGG**GATAAGAAACTCAAATTCACAT	
ClpB DN R	TCAAATTCTTGAGCGCCATC	
Ftsh UP F	AATTTAATAACACGATTCCTGTGACTAAAAAAATGA	CflipH-gfp and NdegH-gfp
Ftsh DN R	TTTAACCTCGCAATTAGATATTCAAGA	
bdSENP1F	**ATTTACCTCCTTTGATTTAA**GTGAATAAGTTCGTCCCAGAACCCTTT	pSENP1
bdSENP1R	AGCTGAATTCCTATCCGGCCTTAAGGTCCAG	
bdNEDP1 F	**ATTTACCTCCTTTGATTTAAGTGAACAAGTTT**ATGGACGAGCGTGTGTTGAGCTAT	pNEDP1
bdNEDP1 R	AGCTGAATTCCTACTGACCACACGTGTCCTCCAC	
Sculp1 F	**ATTTACCTCCTTTGATTTAAGTGAACAAGTTT**ATGAGTAAGCATCATCATCATTCA	pUlp1
Sculp1 R	AGCTGAATTCCTACTTAAGAGCATCCGTTAAAAT	
XYL F	ATTAGGATCCCTAACTTATAGGGGTAACACTTAAAAAAGA	pSENP1, pNEDP1, and pUlp1
XYL R	AAACTTGTTCACTTAAATCAAAGGAGGTAAAT	
Kan F	AGGTGATAGGTAAGATTATACCG	Cflip-bdS, Cflip-bdN, Cflip-scS, Ndeg-bdS, Ndeg-bdN, Ndeg-scS, BDJ2, Cflip-renG, Ndeg-renG, CflipB-gfp, and NdegB-gfp
Kan R	CCCTATCTAGCGAACTTTTAGA	
ldhp F2	TCTAAAAGTTCGCTAGATAGGGTGGTAAATATTAGTGACTTTCTTAACAA	Cflip-bdS, Cflip-bdN, Cflip-scS, Ndeg-bdS, Ndeg-bdN, and Ndeg-scS
ldh UPR2	CGGTATAATCTTACCTATCACCTAGCTGAAAAAGAGCCTTATTTGTGATATA	
ldhp R2	GTTCTAAACATCTCCTTATAATTTATTA	
bdSUMO F	**TAATAAATTATAAGGAGATGTTTAGAAC**ATGTCTGCCGCCGGAGGAGAA	Cflip-bdS and Ndeg-bdS
bdSUMO R1	**ATTCTTTTTAATGATGCCAT**GCCACGTCTTTGGTACAGCAT	
bdSUMO R2	**TAGGAATAGCCTGTGCCCAT**GCCACGTCTTTGGTACAGCAT	
bdNEDD8F	**TAATAAATTATAAGGAGATGTTTAGAAC**ATGATTAAGGTTAAAACACTGAC	Cflip-bdN and Ndeg-bdN
bdNEDD8R1	**ATTCTTTTTAATGATGCCATGC**CACGCAGGGCCAGAA	
bdNEDD8R2	**TAGGAATAGCCTGTGCCCATGCC**ACCACGCAGGGCCAGAA	
ScSUMOF	**TAATAAATTATAAGGAGATGTTTAGAAC**ATGACCGGCAGTGATTCAGAGGTAAAT	Cflip-scS and Ndeg-scS
ScSUMOR1	**ATTCTTTTTAATGATGCCA**CACAAGTCTATTTTCTGTGAG	
ScSUMOR2	**TAGGAATAGCCTGTGCCCAT**CACAAGTCTATTTTCTGTGAG	
HdirC F	ATGGCATCATTAAAAAGAATACGTTAGATACCCCTAT	BDJ2
HdirN F	ATGGGCACAGGCTATTCCTATTTTGGTGAT GGCAAT	
J2UPF	GTTCAAGTTAACAATGGCTTTGATGA	
J2UPR	**CGGTATAATCTTACCTATCACCT**TATGTCTCCTTACATTAGTTTTATAGG	
J2pF	**TCTAAAAGTTCGCTAGATAGGG**CCGTATGTATGAAAGTATTATTATAGT	
J2pR	**GTCAGTGTTTTAACCTTAATCAT**TATGTCTCCTTACATTAGT	
J2F	**TTCTGGCCCTGCGTGGTGGC**ATGAGTGACATTAAAATTATTGCCCTA	
J2R	TTATCTAACTTCCATAACTACTGGTAA	
XYL F2	**TTATAACATGTATTCACGAACGAAAATC**CTAACTTATAGGGGTAACACTTAAAAAAGA	XYLJ2
XYL R2	**TAGGGCAATAATTTTAATGTCACTCAT**AAACTTGTTCACTTAAATCAAAGGAGGTAAAT	
J2 UP R2	**CTAATTGGTAAGCGCGCCATG**TATGTCTCCTTACATTAGTTTTATAGG	
J2 F2	ATGAGTGACATTAAAATTATTGCCCTA	
HdirN-gfp F2	**ATGGCACAGGCTAT**ATGTCAAAAGGAGAAGAGCTGTTC	Ndeg-gfp2
HdirN-gfp F3	**ATGGCACAGGCTATTCCTAT**ATGTCAAAAGGAGAAGAGCTGTTC	Ndeg-gfp3
HdirN-gfp F4	**ATGGGCTATTCCTATTTTGGTGATGGCAATTTTGATACCGTTTTT**ATGTCAAAAGGAGAAGAGCTGTTC	Ndeg-gfp4
HdirC-gfp F3	**ATGACGACTATTAAAAAGCAT**ATGTCAAAAGGAGAAGAGCTGTTC	Cflip-gfp3
HdirC-gfp F2	**ATGACGACTATTAAA**ATGTCAAAAGGAGAAGAGCTGTTC	Cflip-gfp2
HdirC-gfp F4	**ATGACGACTATTAAAAAGCATTTGAGACATCCCTATTGCAAATAAAAAA**TGTCAAAAGGAGAAGAGCTGTTC	Cflip-gfp4
ldhp RN1	**ATAGGAATAGCCTGTGCCAT**GTTCTAAACATCTCCTTATAATTTATTA	Ndeg-gfp2
ldhp RC1	**ATGCTTTTTAATAGTCGTCCTGTGCCAT**GTTCTAAACATCTCCTTATAATTTATTA	Cflip-gfp2
ldhp RN2	**CCTTTTGACATATAGCCTGTGCCATCCAT**GTTCTAAACATCTCCTTATAATTTATTA	Ndeg-gfp3
ldhp RC2	**TTTGACATTTTAATAGTCGTCATAT**GTTCTAAACATCTCCTTATAATTTATTA	Cflip-gfp3
ldhp F3	**TTGAAGAATGAGCAACGTTCTATCTA**ATTATAAGGAGATGTTTAGAACATGAC	Cflip-renG and Ndeg-renG
ldh F2	**TTGAAGAATGAGCAACGTTCTATCTAA**TTATAAGGAGATGTTTAGAACATG	
RenG F	ATGGCTAGTAAAGTTTATGATCCT	
RenG R	TTAGATAGAACGTTGCTCATTCTTCAA	
bdNEDD8R3	**AGGATCATAAACTTTACTAGCCAT**GCCACCACGCAGGGCCAGAA	
bdNEDD8R4	**AGGATCATAAACTTTACTAGCCAT**ATAGGGGTATCTAACGTATTCTTTTTAATGATGCCAT	
XYL R3	TAGGGCAATAATTTTAATGTCACTCATACTTAAATCA	XYLrenG
ldhp F4	**TTGAAGAATGAGCAACGTTCTATCTA**AAGGTAAATATTAGTGACTTTCTTAACAAAAAGT	
Spec F	GATTTTCGTTCGTGAATACATGTTATAA	irvRN-gfp, Cflip-gfp, Cflip-gfp2, Cflip-gfp3, Cflip-gfp4, Ndeg-gfp, Ndeg-gfp2, Ndeg-gfp3, Ndeg-gfp4, XYLrenG, and XYLJ2
Spec R	CATGGCGCGCTTACCAATTAG	

* *Complementary sequences used for overlap extension PCR are shown in bold. Restriction sites are underlined*.

### Strain construction

#### Construction of fluorescent strains

To detect the activity of potential degrons, we appended sequences encoding 11 amino acid candidate degron tags onto the 5′ of the superfolder *gfp* open reading frame (ORF) (Overkamp et al., 2013) and then inserted the tagged *gfp* ORF between the lactate dehydrogenase (*ldh*) promoter and *ldh* ORF using allelic exchange. To generate the constructs, the sequences encoding each of the three tags (HdiR C-flip, Hdir N-degron, and IrvR N-tag) were added to the forward primers of the superfolder *gfp* ORF. The superfolder *gfp* ORF was then amplified from the plasmid pGFPsf (unpublished plasmid) with the forward primers HdirC-gfp F1, HdirN-gfp F1, and irvRN-gfp F1 and the reverse primer gfp R. The *ldh* upstream homologous fragment was PCR amplified from strain UA159 using the primer pair ldh up F/R. The spectinomycin resistance cassette *aad9* was PCR amplified from the shuttle vector pDL278 (Chen and Leblanc, 1992) with the primer pair Spec F/R. The *ldh* promoter fragment was PCR amplified from UA159 gDNA using the primer pairs ldhp F and either ldhp RC1, ldhp RN1, or ldhp RRN1. The *ldh* ORF containing its endogenous Shine-Dalgarno sequence was PCR amplified from strain UA159 using the primer pair ldh F/R. Primers incorporated complementary sequences that facilitated final construct assembly via a 5-fragment overlap extension PCR reaction. The resulting PCR products were transformed into *S. mutans* strain WTLH (Liu et al., 2015) and selected on agar plates supplemented with spectinomycin to obtain the strains Cflip-gfp, Ndeg-gfp, and irvRN-gfp.

To identify the proteases targeted by the HdiR degrons, multiple protease mutants were constructed. The ΔClpB mutation construct was assembled via overlap extension PCR. The upstream homologous fragment of ClpB was PCR amplified from strain UA159 with the primer pair ClpB up F/R. The kanamycin resistance cassette *aphAIII* was PCR amplified from the plasmid pWVKTs (Gutierrez et al., 1996) with the primer pair Kan F/R. The ClpB downstream homologous fragment was PCR amplified with the primer pair ClpB DN F/R. Primers incorporated complementary sequences that facilitated final construct assembly via a 3-fragment overlap extension PCR reaction. The resulting PCR product was transformed into strains Cflip-gfp and Ndeg-gfp and selected on agar plates supplemented with kanamycin to obtain the strains CflipB-gfp and NdegB-gfp, respectively. The ΔClpP mutation was PCR amplified from *S. mutans* strain GN10P (Niu et al., 2010) with the primer pair clpP UP F/clpP DN R. The resulting PCR product was then transformed into *S. mutans* strains Cflip-gfp and Ndeg-gfp and selected on erythromycin to obtain the strains CflipP-gfp and NdegP-gfp. To generate the inducible *ftsH* mutants, the xylose-inducible *ftsH* construct was amplified from strain S1-ftsH (unpublished strain) with the primer pair ftsh UP F/ftsh DN R. The resulting PCR product was transformed into *S. mutans* strains Cflip-gfp and Ndeg-gfp and selected on kanamycin to generate the strains CflipH-gfp and NdegH-gfp.

To compare the effect of different degron tag lengths upon protein degradation, the *gfp* ORF was PCR amplified from strain Cflip-gfp using the primer pairs hdirC-gfp F2, hdirC-gfp F3, or hdirC-gfp F4 together with ldh R. The primer pairs add 4, 6, and 15 amino acid degron tags respectively to the N-terminus of GFP. The same procedure was also repeated using strain Ndeg-gfp and the primer pairs hdirN-gfp F2, hdirN-gfp F3, or hdirN-gfp F4 together with ldh R. The *ldh* promoter was PCR amplified from strain UA159 with the primer pairs ldhp F/ldhp RN(C)1 or ldhp RN(C)2. The *ldh* upstream homologous fragment and spectinomycin resistance cassette *aad9* were PCR amplified from strain Cflip-gfp using the primer pair ldh up F/Spec R. Primers incorporated complementary sequences that facilitated final construct assembly via a 3-fragment overlap extension PCR reaction. The resulting PCR products were transformed into *S. mutans* strain WTLH and selected on agar plates supplemented with spectinomycin to obtain the strains Cflip-gfp2, Cflip-gfp3, Cflip-gfp4, Ndeg-gfp2, Ndeg-gfp3, and Ndeg-gfp4.

To compare the utility of different ubiquitin-like protein/endopeptidase pairs, codon-optimized versions of the UBL proteins (scSUMO, bdSUMO, and bdNEDD8) and their corresponding endopeptidases (scUlp1, bdSENP1, and bdNEDP1) were first synthesized by IDT (Integrated DNA Technologies) (Figure S2). The endopeptidase ORFs were then PCR amplified with three primer pairs: sculp1 F/R, bdSENP1 F/R, and bdNEDP1 F/R. The xylose induction cassette Xyl-S1 was PCR amplified from plasmid pZX9 (Xie et al., 2013) using the primer pair XYL F/R. Primers incorporated complementary sequences as well as *Bam*HI and *Eco*RI restriction sites that facilitated final construct assembly via a 2-fragment overlap extension PCR reaction followed by ligation to the *Bam*HI and *Eco*RI restriction sites of the shuttle vector pDL278 (Chen and Leblanc, 1992). Ligation reactions were transformed into *E. coli* to create the plasmids pUlp1, pSENP1, and pNEDP1. The three plasmids were then transformed into *S. mutans* strain WTLH and selected on agar plates supplemented with spectinomycin to create the *S. mutans* strains smUlp1, smSENP1, and smNEDP1. The UBL proteins were each PCR amplified in two separate reactions with the primer pairs ScSUMO F/ScSUMO R1 or ScSUMO R2, bdSUMO F/bdSUMO R1 or bdSUMO R2, and bdNEDD8 F/bdNEDD8 R1 or bdNEDD8 R2. The kanamycin resistance cassette *aphAIII* was amplified from the plasmid pWVKTs with the primer pair Kan F/R. Degron tagged *gfp* and the downstream *ldh* ORF were PCR amplified from *S. mutans* strains Cflip-gfp and Ndeg-gfp using the primer pairs HdirC F/ldh R and HdirN F/ldh R. The *ldh* promoter was PCR amplified from strain UA159 using the primer pair ldhp F2/ldhp R2. The *ldh* upstream homologous fragment was PCR amplified from UA159 using the primer pair ldh UP F/ldh UP R2. Primers incorporated complementary sequences that facilitated final construct assembly via a 5-fragment overlap extension PCR reaction. The resulting PCR products were transformed into *S. mutans* strains smUlp1, smSENP1, and smNEDP1 and selected on agar plates supplemented with kanamycin to obtain the strains Cflip-scS, Ndeg-scS, Cflip-bdS, Ndeg-bdS, Cflip-bdN, and Ndeg-bdN.

#### Construction of luciferase reporters

To compare the depletion kinetics of targeted proteolysis vs. transcriptional depletion, the green renilla luciferase enzyme was targeted using both depletion strategies. The *renG* ORF containing a Shine Dalgarno sequence was PCR amplified from *S. mutans* strain iRenGIV (Merritt et al., 2016) with the primer pair RenG F/R. DNA fragments encoding the *ldh* promoter and both of the hybrid bdNEDD8+HdiR degron fusion tags were PCR amplified from strains Cflip-bdN and Ndeg-bdN using the primer pairs ldhp F2/bdNEDD8R3 or bdNEDD8R4. The *ldh* ORF containing its endogenous Shine-Dalgarno sequence was PCR amplified from strain UA159 with the primers ldh F2/R. The *ldh* upstream homologous fragment and kanamycin resistance cassette *aphAIII* were PCR amplified from *S. mutans* strain Cflip-bdN using the primers ldh UP F/Kan R. Primers incorporated complementary sequences that facilitated final construct assembly via a 4-fragment overlap extension PCR reaction. The resulting PCR products were transformed into *S. mutans* strain smNEDP1 and selected on agar plates supplemented with kanamycin to obtain the strains Cflip-renG and Ndeg-renG. To create a xylose-inducible green renilla luciferase reporter strain, the *ldh* upstream homologous region, spectinomycin resistance cassette *aad9*, and the xylose induction cassette Xyl-S1 were PCR amplified from the *S. mutans* strain XYLJ2 using the primer pair ldh UP F/XYLR3. The *renG* ORF was PCR amplified from *S. mutans* strain iRenGIV using the primer pair RenG F/R. The *ldh* promoter region and ORF was PCR amplified from strain UA159 with the primer pair ldhp F4/ldh R. Primers incorporated complementary sequences that facilitated final construct assembly via a 3-fragment overlap extension PCR reaction. The resulting PCR product was transformed into strain UA159 and selected on agar plates supplemented with spectinomycin to obtain the strain XYLrenG.

#### Construction of RNase J2 depletion strains

To compare the efficiency of targeted protein degradation vs. transcriptional depletion for the study of toxic mutations, the pleiotropic regulator RNase J2 from *S. mutans* was targeted using both depletion strategies. A DNA fragment encoding the hybrid bdNEDD8+C-flip degron fusion tag was PCR amplified from *S. mutans* strain Cflip-bdN using the primer pair bdNEDD8F/bdNEDD8R1. The kanamycin resistance cassette *aphAIII* was PCR amplified from the plasmid pWVKTs with the primer pair Kan F/R. The promoter region of RNase J2 was PCR amplified from strain UA159 using the primer pair J2P F/R. The RNase J2 ORF with endogenous Shine-Dalgarno sequence was PCR amplified from strain UA159 using the primer pair J2 F/R. The RNase J2 upstream homologous fragment was PCR amplified from strain UA159 with the primer pair J2 UP F/R. Primers incorporated complementary sequences that facilitated final construct assembly via a 5-fragment overlap extension PCR reaction. The resulting PCR product was transformed into *S. mutans* strain smNEDP1 and selected on agar plates supplemented with kanamycin to obtain the strain bdNJ2. To create a xylose-inducible RNase J2 strain, the RNase J2 ORF was transcriptionally fused to the Xyl-S1 induction cassette. Xyl-S1 was PCR amplified from plasmid pZX9 using the primer pair XYL F2/R2, while the spectinomycin resistance cassette *aad9* was PCR amplified from plasmid pDL278 using the primers Spec F/R. The RNase J2 upstream homologous fragment and the RNase J2 ORF were both PCR amplified from strain UA159 using the primer pairs J2 UP F/R2 and J2 F2/R, respectively. The primers incorporated complementary sequences that facilitated final construct assembly via a 4-fragment overlap extension PCR reaction. The resulting PCR products were transformed into *S. mutans* strain WTLH and selected on agar plates supplemented with spectinomycin to obtain the *S. mutans* strain XYLJ2.

### Immunodetection of epitope tagged proteins

The preparation of cell lysates and western blots was performed similarly as previously described (Liu et al., 2015). α-FLAG and α-HA rabbit primary antibodies (ThermoFisher Scientific) were both diluted to 1:2,000, while HRP- conjugated goat α-rabbit IgG secondary antibody (ThermoFisher Scientific) was diluted 1:10,000. HA tagged lactate dehydrogenase (Ldh) served as a loading control for all western blots. One micrograms total protein was loaded for GFP and Ldh western blots, while 5 μg total protein was loaded for RNase J2 western blots.

### *S. mutans* microscopy

The superfolder variant of green fluorescent protein (GFP) was used to assess *S. mutans* degron activity *in situ*. Overnight cultures were diluted 1:30 and grown to mid-log phase before imaging with both differential interference contrast microscopy and epifluorescence microscopy. Images were captured using oil immersion at 100x magnification (1000x total) with an Olympus IX73 inverted epifluorescence microscope and attached Olympus XM10 camera. All fluorescent images were exposed for 90 ms. with identical settings and processing using Olympus cellSens software ver. 1.11. All strains and growth conditions assayed via microscopy were imaged using a minimum of three separate fields. Representative images were presented.

### Luciferase activity assay

Overnight cultures were diluted 1:30 into fresh THYE medium and grown for 3 h. to an optical density OD_600_ 0.2–0.3. To compare targeted proteolysis vs. transcriptional depletion, xylose-inducible luciferase cultures also contained 1% (wt/vol) xylose to maintain the production of luciferase during the initial incubation period. At T_0_, 1% (wt/vol) xylose inducer was added to the degron tagged luciferase strains, whereas xylose was removed from the inducible luciferase strain via washout. The cultures were then incubated over a time course for a total of 3 h. To assess the population-level tunability of the targeted proteolysis system, degron tagged luciferase strains were cultured in the presence of a range of xylose concentrations for a total of 3 h. before measuring luciferase activity. Luciferase activity was measured similarly as previously described (Merritt et al., 2016) using a Promega GloMAX (Promega). Luciferase activity was normalized to the optical density of the cultures, which was measured using a Biospectrometer (Eppendorf). All luciferase data are presented as the average values from three independent experiments.

### Dextran-dependent aggregation (DDAG)

Overnight cultures were diluted (1:30) into fresh THYE medium and grown for 3 h. to an optical density OD_600_ 0.2–0.3. Xylose-inducible RNase J2 cultures also contained 1% (wt/vol) xylose to maintain the production of RNase J2 during the initial incubation period. At T_0_, 1% (wt/vol) xylose inducer was added to the degron tagged RNase J2 strain, whereas xylose was removed from the inducible RNase J2 strain via washout. The cultures were then incubated for over a time course for a total of 3 h. At each time point, 100 μg ml^−1^ dextran T2000 from *Leuconostoc* spp. (Sigma) was added into the culture tubes and then swirled briefly to mix. Visible aggregation was observed within 1–2 min. of dextran addition.

## Results

### LexA-like proteins encode embedded N- and C-terminal degrons

**Figure 1:**
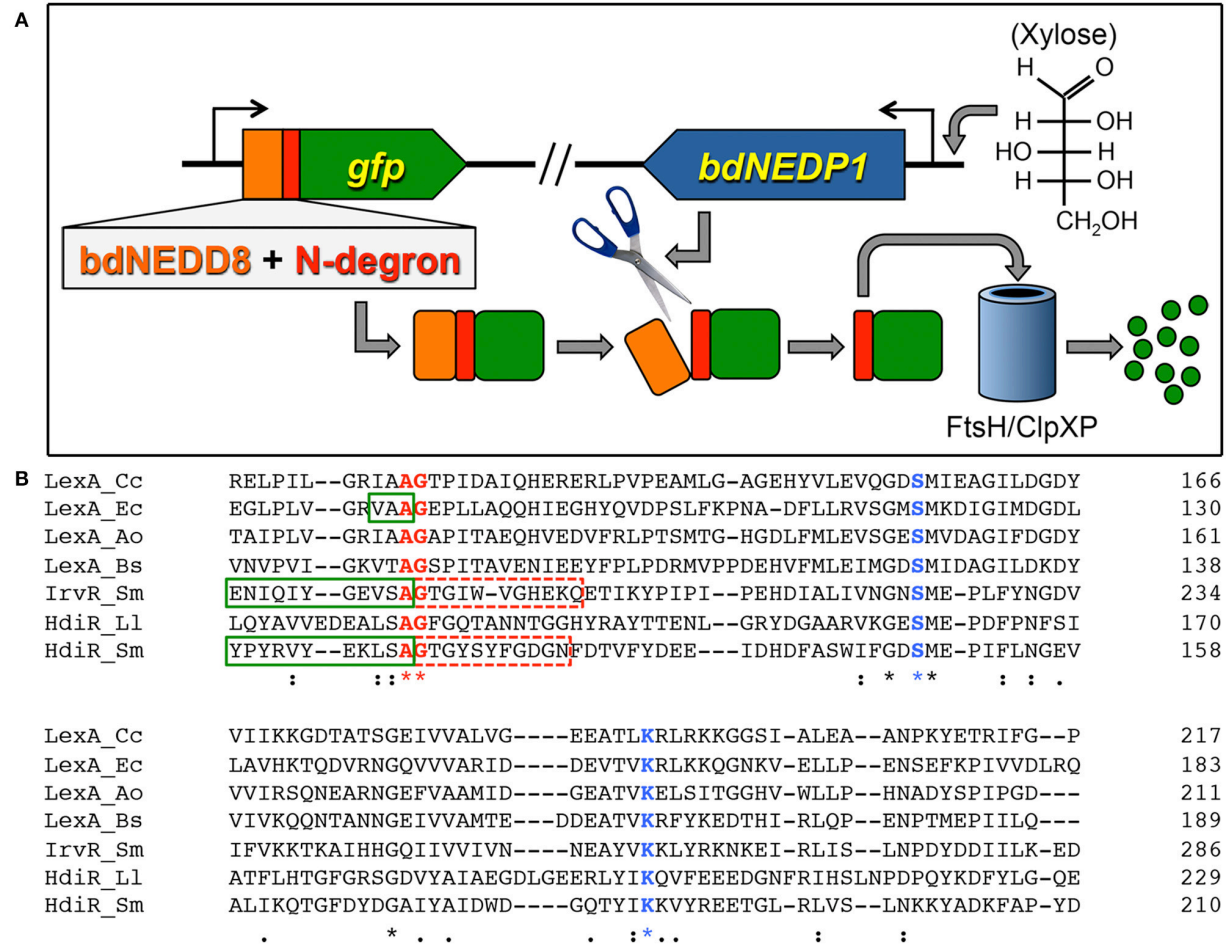
Controllable proteolysis with HdiR degrons. **(A)** Overview of the controllable N-terminal proteolysis system. The bdNEDD8 tag is represented in orange, while the N-degron is represented in red. **(B)** A phylogenetically diverse group of LexA orthologs and LexA-like proteins were aligned using the CLUSTAL Omega webserver (https://www.ebi.ac.uk/Tools/msa/clustalo/). Conserved residues are indicated by asterisks beneath their respective amino acids. Conserved residues shown in blue font are catalytic site residues of S24 peptidase domains, while residues in shown in red font comprise the autocleavage sites. Green boxes are drawn around confirmed C-degrons. Dashed red boxes indicate residues tested for N-degron activity. Species names are abbreviated as follows: Cc, *Caulobacter crescentus*; Ec, *Escherichia coli*; Ao, *Actinomyces odontolyticus*; Bs, *Bacillus subtilis*; Sm, *Streptococcus mutans*; Ll, *Lactococcus lactis*.

**Figure 2:**
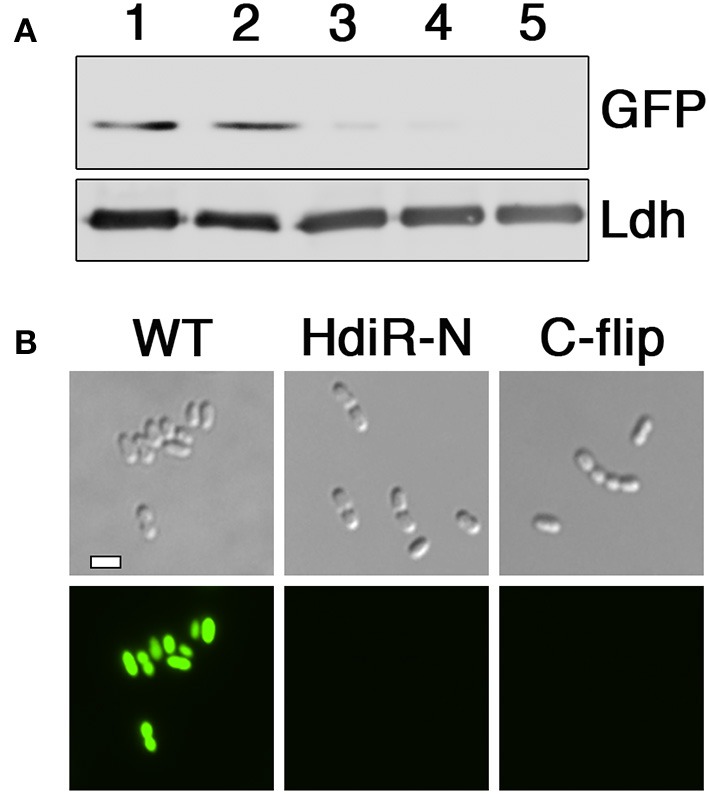
Determination of N-degron activity from HdiR autocleavage site epitopes. **(A)** Peptide tags derived from the autocleavage site of HdiR were appended onto the N-terminus of a constitutively expressed superfolder variant of the green fluorescent protein (GFP) harboring a C-terminal 3x FLAG tag. GFP abundance was compared via western blot using α-FLAG primary antibody. The *S. mutans* lactate dehydrogenase (Ldh) was used as a loading control. Samples from left to right are as follows: 1 (GFP), 2 (IrvR N-epitope tagged GFP), 3 (HdiR N-degron tagged GFP), 4 (HdiR C-flip degron tagged GFP), and 5 (GFP lacking a 3x FLAG epitope). **(B)** Cells expressing GFP containing either no degron (WT), the HdiR N-degron (HdiR-N), or the HdiR C-flip degron (C-flip) were imaged using both differential interference contrast microscopy (top) and epifluorescence microscopy with a 90 ms. exposure time (bottom). Scalebar indicates 1 μm.

An overview of the N-terminal targeted proteolysis system is presented in Figure 1A. As previously described, a small number of controllable proteolysis systems have been developed for use in some Gram positive and Gram negative bacteria. All of these systems exploit the SsrA C-degron encoded by the tmRNA to trigger proteolytic degradation of the proteins of interest (McGinness et al., 2006; Griffith and Grossman, 2008; Davis et al., 2011; Kim et al., 2011; Wei et al., 2011; Cameron and Collins, 2014). The SsrA C-degron is particularly useful because its sequence can be easily identified within the tmRNA of different species. Since this degron is only functional when exposed at the C-terminus (Wei et al., 2011), all of the current targeted proteolysis systems are limited to C-terminal tagging. This may be problematic for some proteins if, for example, they contain extracytoplasmic C-termini or require their C-termini for functionality or protein-protein interactions. In which case, it would be highly desirable to target proteins via their N-termini. Unfortunately, of the small number of SsrA-independent degrons reported in the literature, nearly all are C-degrons. Surprisingly little is known about prokaryotic N-degrons. In some bacteria like *E. coli*, N-degrons are subject to the N-end rule and require the adaptor protein ClpS (Dougan et al., 2010). However, the N-end rule pathway is still poorly understood and it is unclear whether these rules apply universally to all bacteria. Furthermore, we were unable to detect obvious ClpS homologs in *S. mutans* or *Bacillus subtilis* (data not shown). In our previous studies, we identified an 11 amino acid sequence within the LexA-like transcription repressor IrvR that becomes exposed as a ClpXP-dependent C-degron following IrvR autocleavage cleavage into two fragments (Niu et al., 2010). In fact, a similarly buried ClpXP-dependent C-degron is also a key feature of the *E. coli* LexA (Neher et al., 2003). LexA and LexA-like proteins encode these embedded degrons directly adjacent to their strictly conserved autocleavage sites (Figure 1B) to trigger rapid degradation of their N-terminal DNA-binding domains immediately following SOS-induced autocleavage. However, it was unknown whether embedded N-degrons were also present to degrade the C-terminal domains of LexA-like proteins following autocleavage. To test this, we tagged the N-terminus of GFP with the first 11 amino acids after the autocleavage sites of both IrvR (SMU_1398; GTGIWVGHEKQ) and the *S. mutans* LexA-like regulator HdiR (SMU_2027; GTGYSYFGDGN) (Savijoki et al., 2003; Varhimo et al., 2007; Boutry et al., 2013; Leung et al., 2015; Figure 1B) to determine whether they would confer constitutive proteolysis. While we did not detect any N-degron activity from the IrvR epitope (data not shown), the HdiR epitope exhibited extremely potent N-degron activity (Figures 2A,B). As a potential approach to identify additional N-degrons, we were curious whether it would be possible to invert the endogenous HdiR C-degron sequence (Figure 1B) and create a viable N-degron. To our great surprise, this inverted degron, which we refer to as “C-flip” performed as well as the endogenous HdiR N-degron to target GFP for constitutive proteolysis (Figures 2A,B). It is also worth noting that GFP was expressed as a transcriptional fusion to the *S. mutans* lactate dehydrogenase, which is among the highest expressed constitutive genes in this organism (Svensater et al., 2001; Merritt et al., 2005). Therefore, both HdiR degron tags must confer exceptionally rapid and efficient degradation, as the depletion of the highly stable superfolder GFP variant (Pedelacq et al., 2006; Overkamp et al., 2013) employed in these experiments clearly outpaced its synthesis (Figures 2A,B).

### Characterization of the HdiR N- and C-flip degrons

**Figure 3:**
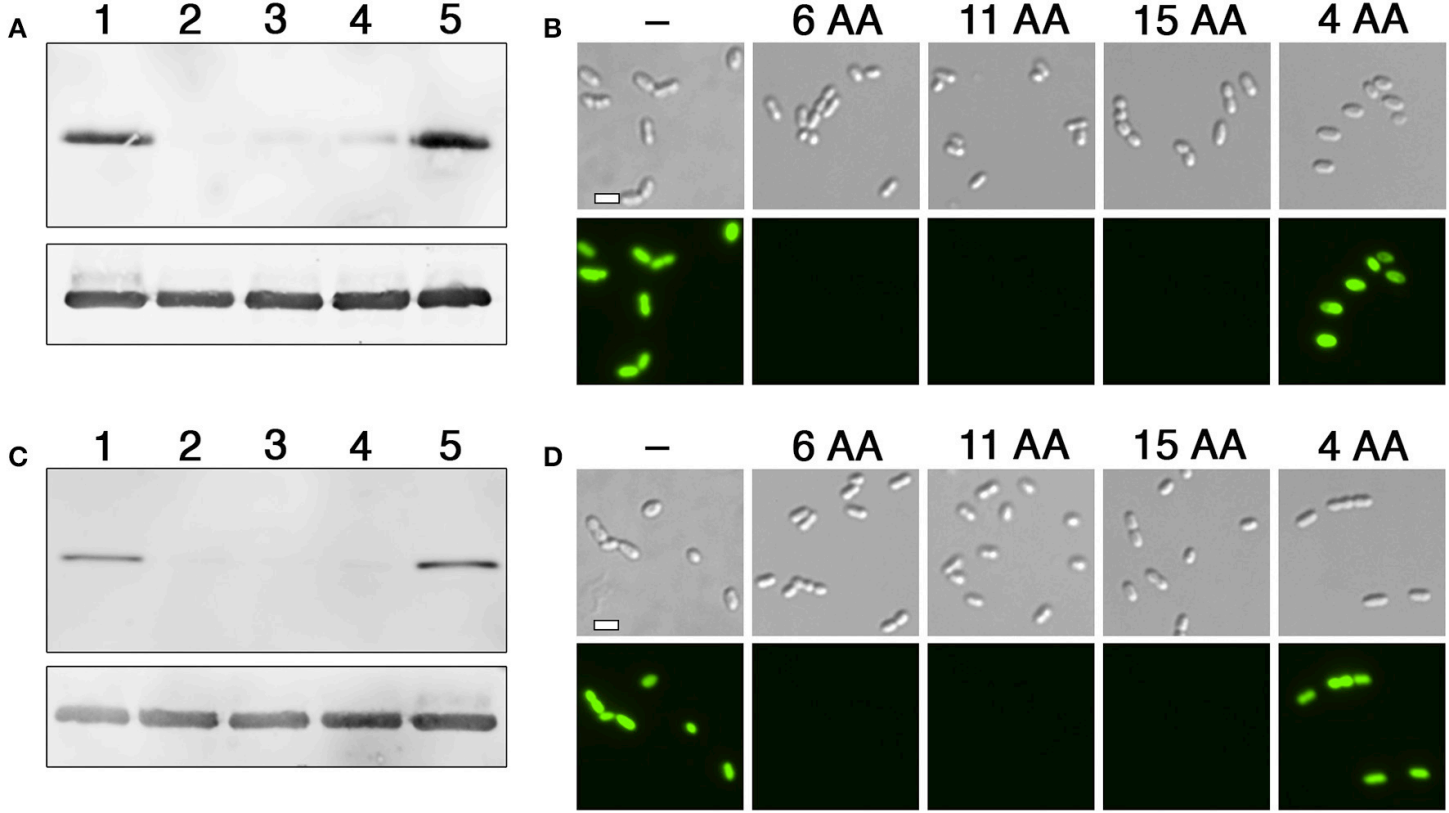
Comparison of degron activity using different sized tags. **(A)** HdiR N-degron tags of varying sizes were appended onto the N-terminus of superfolder GFP and examined via western blot. Samples from left to right: 1 (no degron tag), 2 (GTGYSY degron tag), 3 (GTGYSYFGDGN degron tag), 4 (GTGYSYFGDGNFDTV degron tag), and 5 (GTGY degron tag). The *S. mutans* lactate dehydrogenase was used as a loading control (bottom). **(B)** The same strains were imaged using both differential interference contrast microscopy (top) and epifluorescence microscopy with a 90 ms. exposure time (bottom). The numbers of amino acids (AA) included in the degron tags are indicated above the respective images. **(C)** HdiR C-flip degron tags of varying sizes were appended onto the N-terminus of superfolder GFP and examined via western blot. Samples from left to right: 1 (no degron tag), 2 (ASLKEY degron tag), 3 (ASLKEYVRYPY degron tag), 4 (ASLKEYVRYPYRKNK degron tag), and 5 (ASLK degron tag). The *S. mutans* lactate dehydrogenase was used as a loading control (bottom). **(D)** The same strains were imaged using both differential interference contrast microscopy (top) and epifluorescence microscopy with a 90 ms. exposure time (bottom). The numbers of amino acids (AA) included in the degron tags are indicated above the respective images. Scalebars indicate 1 μm.

**Figure 4:**
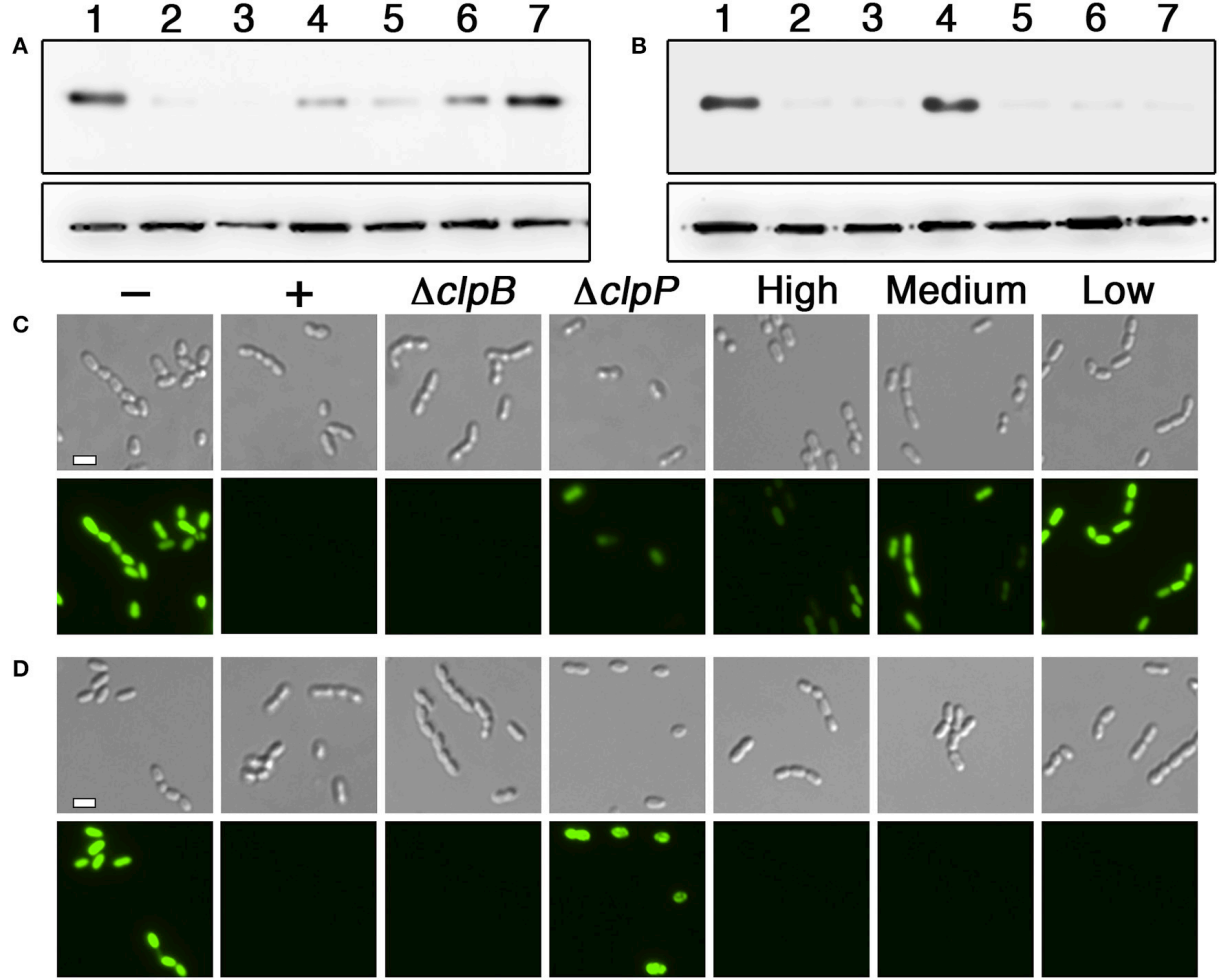
Determination of the proteases targeted by HdiR degrons. **(A)** The HdiR N-degron tag was appended onto the N-terminus of superfolder GFP and examined via western blot. Samples from left to right: 1 (no degron tag), 2 (HdiR N-degron tag), 3 (HdiR N-degron tag + a mutation of *clpB*), 4 (Hdir N-degron tag + a mutation of *clpP*), 5 (HdiR N-degron tag + 0.5% wt/vol xylose induction of *ftsH*), 6 (HdiR N-degron tag + 0.1% wt/vol xylose induction of *ftsH*), and 7 (HdiR N-degron tag + 0.05% wt/vol xylose induction of *ftsH*). The *S. mutans* lactate dehydrogenase was used as a loading control (bottom). **(B)** The same experiment was repeated, except the HdiR C-flip degron tag was used. **(C)** The HdiR N-degron tag was appended onto the N-terminus of superfolder GFP and imaged using both differential interference contrast microscopy (top) and epifluorescence microscopy with a 90 ms. exposure time (bottom). Samples from left to right: “–” (no degron tag), “+” (HdiR N-degron tag), “Δ*clpB*” (HdiR N-degron tag + a mutation of *clpB*), “Δ*clpP*” (HdiR N-degron tag + a mutation of *clpP*), “High” (HdiR N-degron tag + 0.5% wt/vol xylose induction of *ftsH*), “Medium” (HdiR N-degron tag + 0.1% wt/vol xylose induction of *ftsH*), and “Low” (HdiR N-degron tag + 0.05% wt/vol xylose induction of *ftsH*). D) The same experiment was repeated, except the HdiR C-flip degron tag was used. Scalebars indicate 1 μm.

To identify the minimal degron for both tags, we tested a variety of degron lengths and compared their efficiencies. For both the N- and C-flip degrons, we found similarly potent activity when using tags containing between 6 and 15 amino acids adjacent to the HdiR autocleavage site, whereas their activity was completely abolished with 4 amino acid tags (Figures 3A–D). Therefore, the minimal N-degron length is likely between 5 and 6 amino acids from the autocleavage site. To ensure reliable degron activity for most proteins, we proceeded with 11 amino acid degron tags, as we previously observed that the minimal IrvR C-degron functions well as a tag for some proteins, while others require longer tags to trigger proteolysis (Niu et al., 2010). Given the potent activity of both HdiR degrons, we next deleted a variety of housekeeping proteases to determine which mutations might suppress their constitutive degradation phenotypes. Since we determined an *ftsH* mutation to be lethal, it was necessary to test this protease using a transcriptional depletion approach (Figure S1). Interestingly, the HdiR N- and C-flip degrons exhibited distinct targeting abilities. Both western blot and microscopy data suggested that the HdiR N-degron primarily targets FtsH, as there was a strong inverse correlation between *ftsH* expression and the abundance of GFP in the cell (Figures 4A,C). This degron also exhibits a weak affinity for ClpP-dependent proteases, indicating that the HdiR N-degron likely exhibits some promiscuity. This is in contrast to the C-flip degron, which exclusively targets the ClpP-dependent proteases (Figures 4B,D) much like its original parent C-degron (Frees et al., 2007).

### Creation of an exogenously controlled N-terminal proteolysis system

**Figure 5:**
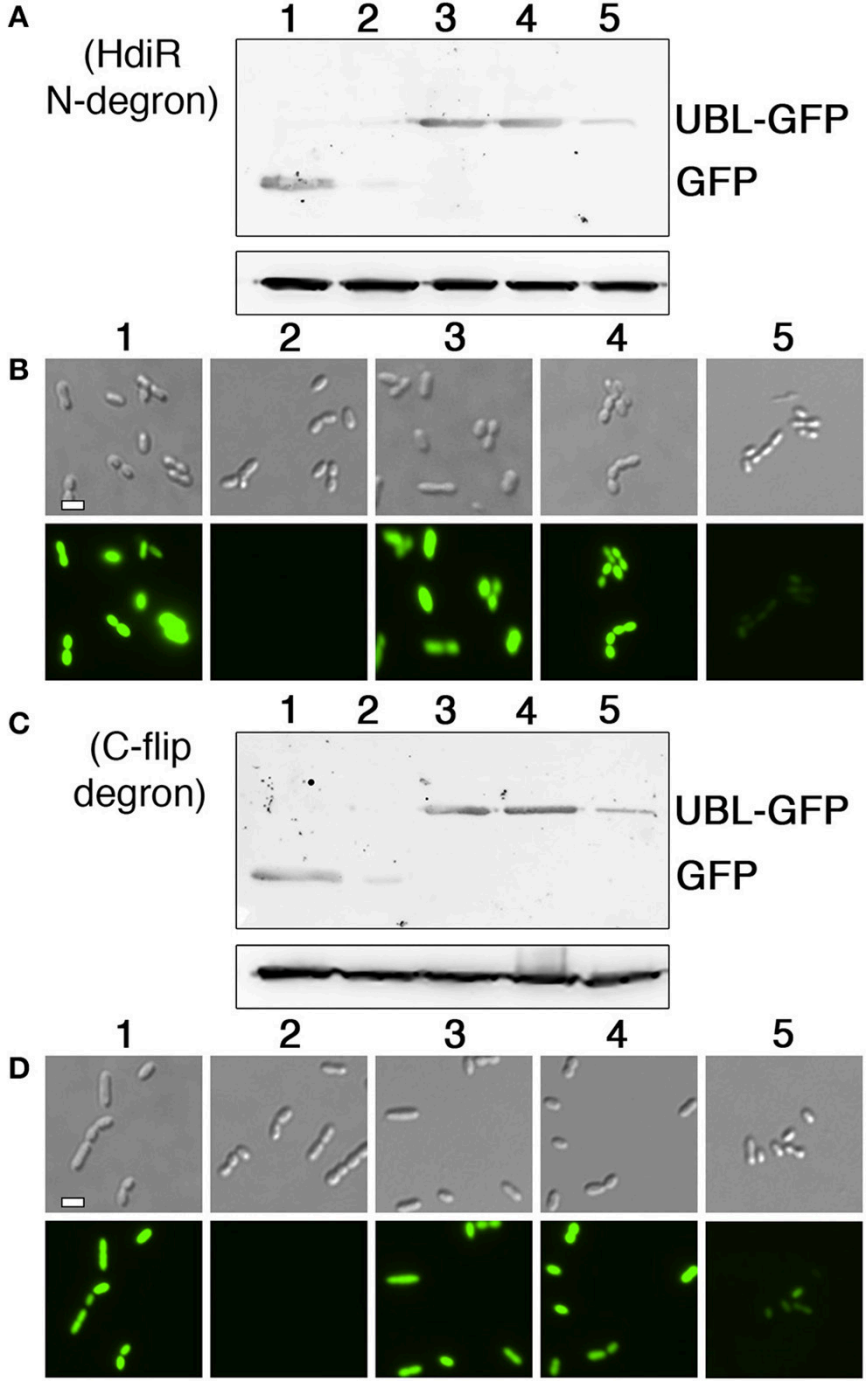
Comparison of ubiquitin-like protein tag/endopeptidase pairs for controlled proteolysis. **(A)** The HdiR N-degron was fused to the C-termini of three different ubiquitin-like protein tags and then each hybrid tag was appended onto the N-terminus of superfolder GFP. The corresponding endopeptidases for each ubiquitin-like protein were expressed from a xylose-inducible promoter. Protein abundance was assessed via western blot following 1 h. xylose induction. The *S. mutans* lactate dehydrogenase was used as a loading control (bottom). Samples from left to right: 1 (no degron tag), 2 (HdiR N-degron tag), 3 (bdSUMO+HdiR N-degron tag with bdSENP1 endopeptidase), 4 (scSUMO+HdiR N-degron tag with scUlp1 endopeptidase), and 5 (bdNEDD8+HdiR N-degron tag with bdNEDP1 endopeptidase). **(B)** The same samples were also imaged using both differential interference contrast microscopy (top) and epifluorescence microscopy with a 90 ms. exposure time (bottom). **(C)** The HdiR C-flip degron was fused to the C-termini of three different ubiquitin-like protein tags and then each hybrid tag was appended onto the N-terminus of superfolder GFP. The corresponding endopeptidases for each ubiquitin-like protein were expressed from a xylose-inducible promoter. Protein abundance was assessed via western blot following 1 h. xylose induction. The *S. mutans* lactate dehydrogenase was used as a loading control (bottom). Samples from left to right: 1 (no degron tag), 2 (HdiR C-flip degron tag), 3 (bdSUMO+HdiR C-flip degron tag with bdSENP1 endopeptidase), 4 (scSUMO+HdiR C-flip degron tag with scUlp1 endopeptidase), and 5 (bdNEDD8+HdiR C-flip degron tag with bdNEDP1 endopeptidase). **(D)** The same samples were also imaged using both differential interference contrast microscopy (top) and epifluorescence microscopy with a 90 ms. exposure time (bottom). Scalebars indicate 1 μm.

**Figure 6:**
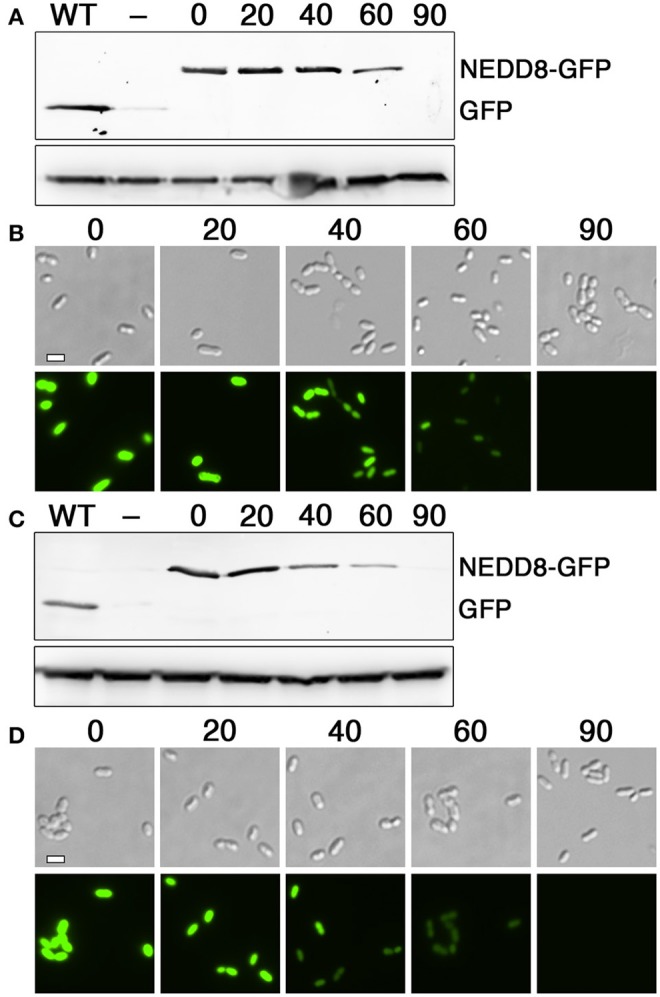
Optimization of bdNEDP1 induction time. **(A)** The hybrid bdNEDD8+HdiR N-degron tag was appended onto the N-terminus of superfolder GFP and then analyzed via western blot after inducing bdNEDP1 expression over a timecourse. The *S. mutans* lactate dehydrogenase was used as a loading control (bottom). Samples from left to right: “WT” (no degron tag), “–” (HdiR N-degron tag), “0” (bdNEDD8+HdiR N-degron tag), “20” (bdNEDD8+HdiR N-degron tag after 20 min. bdNEDP1 induction), “40” (bdNEDD8+HdiR N-degron tag after 40 min. bdNEDP1 induction), “60” (bdNEDD8+HdiR N-degron tag after 60 min. bdNEDP1 induction), and “90” (bdNEDD8+HdiR N-degron tag after 90 min. bdNEDP1 induction). **(B)** Superfolder GFP containing the hybrid bdNEDD8+HdiR N-degron tag was imaged using both differential interference contrast microscopy (top) and epifluorescence microscopy with a 90 ms. exposure time (bottom). The numbers above the images indicate bdNEDP1 induction times (min.). **(C)** The hybrid bdNEDD8+HdiR C-flip degron tag was appended onto the N-terminus of superfolder GFP and then analyzed via western blot after inducing bdNEDP1 expression over a timecourse. The *S. mutans* lactate dehydrogenase was used as a loading control (bottom). Samples from left to right: “WT” (no degron tag), “−” (HdiR C-flip degron tag), “0” (bdNEDD8+HdiR C-flip degron tag), “20” (bdNEDD8+HdiR C-flip degron tag after 20 min. bdNEDP1 induction), “40” (bdNEDD8+HdiR C-flip degron tag after 40 min. bdNEDP1 induction), “60” (bdNEDD8+HdiR C-flip degron tag after 60 min. bdNEDP1 induction), and “90” (bdNEDD8+HdiR C-flip degron tag after 90 min. bdNEDP1 induction). **(D)** Superfolder GFP containing the hybrid bdNEDD8+HdiR C-flip degron tag was imaged using both differential interference contrast microscopy (top) and epifluorescence microscopy with a 90 ms. exposure time (bottom). The numbers above the images indicate bdNEDP1 induction times (min).

**Figure 7:**
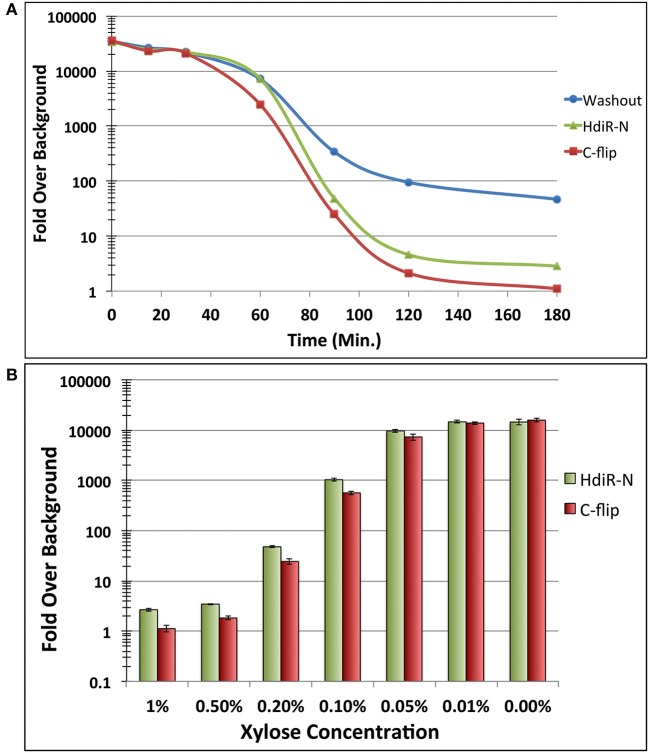
Degradation kinetics and tunability of targeted proteolysis. **(A)** The two hybrid bdNEDD8+HdiR degron tags were appended onto the N-termini of green renilla luciferase to compare their performance relative to transcriptional depletion of a xylose-inducible luciferase. At T_0_, inducer was added to both of the degron tagged strains to initiate the proteolysis of luciferase. Simultaneously, inducer was removed from the inducible luciferase strain via washout to initiate its transcriptional depletion. Data are presented as the ratio of normalized luminescence intensity values to that of the background luminescence (i.e., fold over background). Values represent the means of 3 independent experiments. Standard deviations for all data points are <10%. **(B)** The same degron tagged luciferase strains were both grown for 3 h. in the presence of a range of inducer concentrations to assess the population-level tunability of the system. Data are presented as the ratio of normalized luminescence intensity values to that of the background luminescence. Values represent the means of three independent experiments ± standard deviations.

**Figure 8:**
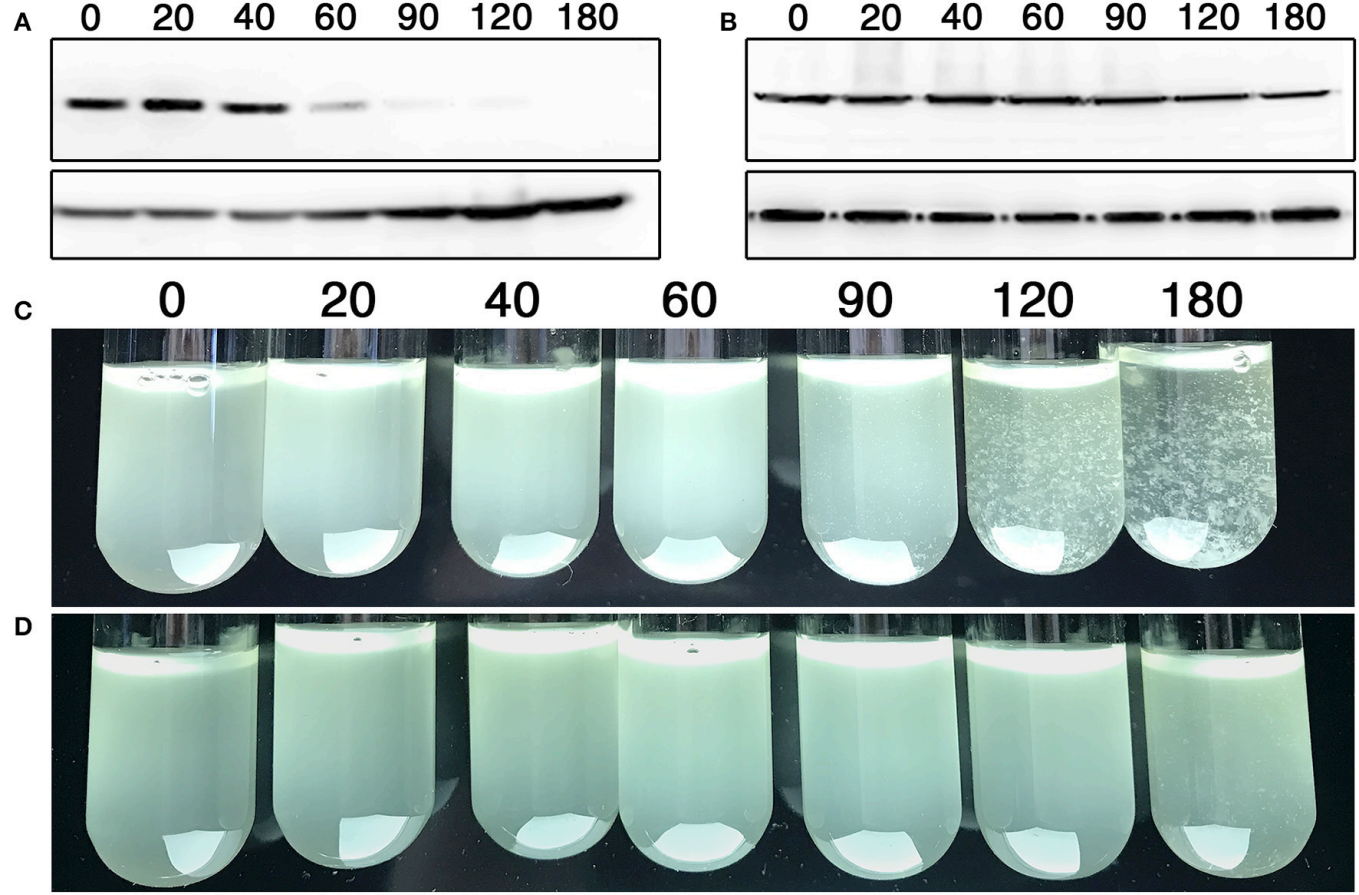
Targeted proteolysis vs. transcriptional depletion of the pleiotropic regulator RNase J2. **(A)** The hybrid bdNEDD8+HdiR C-flip degron tag was appended onto the N-terminus of RNase J2 and then analyzed via western blot after inducing bdNEDP1 expression over a timecourse. The *S. mutans* lactate dehydrogenase was used as a loading control (bottom). Time (min) following bdNEDP1 induction is shown above the respective samples. **(B)** A xylose-inducible RNase J2 expression strain was analyzed via western blot after initiating transcriptional depletion via inducer washout. The *S. mutans* lactate dehydrogenase was used as a loading control (bottom). Time (min.) following inducer washout is shown above the respective samples. **(C)** The hybrid bdNEDD8+HdiR C-flip degron tag was appended onto the N-terminus of RNase J2 and subsequently examined for aggregation after inducing bdNEDP1 expression over a timecourse. Time (min.) following bdNEDP1 induction is shown above the respective samples. **(D)** Cultures of a xylose-inducible RNase J2 expression strain were examined for aggregation after initiating transcriptional depletion via inducer washout. Time (min) following inducer washout is shown above the respective samples.

HdiR and other LexA-like proteins are only destabilized following endoproteolytic autocleavage (Neher et al., 2003; Frees et al., 2007; Niu et al., 2010), which strongly suggests that the endogenous N- and C-degrons embedded within HdiR only function when exposed at their respective N- and C-termini. Therefore, we reasoned it should be feasible to engineer an analogous two-step degradation mechanism into heterologous proteins. Since the HdiR degrons mediate the second step of the degradation mechanism, we were interested to next identify an endopeptidase cleavage epitope that could be used to mask the degrons until the appropriate endopeptidase is produced. We examined numerous potential endopeptidases to identify those meeting two criteria: (1) they have a well-defined and strict cleavage specificity and (2) they cut on the C-terminal side of their consensus cut sites. Some of the most promising candidates were identified among the small ubiquitin-like (UBL) proteins of eukaryotes, which play key roles as posttranslational modifiers of protein function and stability in numerous pathways (Kerscher et al., 2006). UBL proteins such as SUMO and NEDD8 utilize extremely specific endopeptidases to activate them for conjugation to target proteins (Shen et al., 2005). Recently, a variety of UBL protein/endopeptidase pairs from various species were tested in *E. coli* for their potential utility as cleavable protein purification tags and found to greatly outperform traditionally used systems, such as the Tobacco Etch Virus (TEV) protease (Frey and Gorlich, 2014). Thus, we selected the 3 best performing enzymes from this study (scUlp1, bdSENP1, and bdNEDP1) and synthesized codon-optimized versions of the UBL protein tags and their respective endopeptidases (Figure S2). The UBL protein tags were appended onto the N-terminus of degron-tagged GFP and their respective endopeptidases were placed under the control of a xylose-inducible promoter. In the absence of induction, all of the GFP fusion constructs fluoresced indistinguishably from the unmodified GFP (data not shown). However, after 1 h. of xylose induction, there was substantially reduced GFP abundance from both of the strains harboring an HdiR degron and the bdNEDD8/bdNEDP1 UBL protein/endopeptidase pair, whereas no obvious GFP degradation was triggered by the scSUMO/scUlp1 and bdSUMO/bdSENP1 pairs (Figures 5A–D). Since GFP degradation with the bdNEDD8/bdNEDP1 pair was not as complete as previously observed (Figures 2A,B), we next tested a range of bdNEDP1 induction times. Consistent with our previous results, 60 min. bdNEDP1 induction was sufficient to remove most of the GFP in the cell, but not all. Noticeable reductions in GFP abundance were apparent by 40 min. post-induction, and by 90 min., GFP was nearly undetectable (Figures 6A–D). Interestingly, the microscopy results indicated that GFP degradation kinetics are not homogenous among the population. Some cells respond earlier than other others, presumably as a stochastic event (Figures 6B,D). This may be due to asynchronous gene induction of bdNEDP1 within the population (Morgan-Kiss et al., 2002). As mentioned previously, transcriptional depletion is currently the standard approach to study essential genes and otherwise toxic mutations. Therefore, it was of interest to determine how our proteolysis system would compare to a typical transcriptional depletion approach. To test this, we created N-terminal luciferase fusions using both of the hybrid bdNEDD8+HdiR degron tags and compared their degradation kinetics with that of a transcriptionally depleted, inducible luciferase construct. Luciferase was chosen due to its low intrinsic stability at 37°C (Loening et al., 2006; Branchini et al., 2007; Ebrahimi et al., 2012; Rahnama et al., 2017), which provides a best-case scenario to reduce protein abundance via transcriptional depletion. At T_0_, we added xylose to the degradable luciferase strains to trigger bdNEDP1 expression, while simultaneously removing xylose from the inducible luciferase culture (i.e., washout) to initiate its transcriptional depletion. As shown in Figure 7A, we achieved >4 orders of magnitude reduction in luciferase activity from both of the degradable luciferase strains. The HdiR C-flip degron initiated degradation slightly earlier than the N-degron and resulted in a total loss of detectable luciferase signal by 180 min. post-induction, whereas there was a small, but detectable amount of signal left for the N-degron tagged enzyme. In stark contrast, the transcriptional depletion approach was both much slower and far less efficient than both of the degron targeted luciferases. By 180 min. after washout, the inducible luciferase strain yielded 20- and 45-fold more luciferase activity compared to the HdiR N-degron and C-flip degron strains, respectively (Figure 7A). Using both of the degron tagged luciferases, we also tested a range of xylose concentrations to assess the population-level tunability of the targeted proteolysis system. At inducer concentrations ranging from 0.01 to 1% (wt/vol) xylose, we could modulate the luciferase activities of the strains throughout the entirety of their 4-log range of potential luciferase values (Figure 7B). Thus, the system offers an exceptionally wide range of possible protein concentrations that can be manually selected according to the amount of inducer added to the cultures. As a final comparison, we fused the hybrid bdNEDD8+C-flip degradation tag onto the N-terminus of RNase J2, which is a pleiotropic housekeeping regulatory RNase of *S. mutans*. An RNase J2 mutant exhibits severe growth defects (Chen et al., 2015) as well as a vigorous aggregation phenotype in liquid culture due to its inability to degrade *gbpC* mRNA, which results in the overexpression of the GbpC surface adhesin (Liu et al., 2015). Similar to the previous GFP experiments, by 90 min. after bdNEDP1 induction, RNase J2 was only weakly detectable by western blot (Figure 8A). This correlated with the first signs of aggregation in liquid culture and by 180 min. post-induction, the aggregation phenotype was indistinguishable from that of an RNase J2 mutant (Figure 8C; Liu et al., 2015). Unexpectedly, we also noted a clear inverse correlation between RNase J2 protein abundance and that of our lactate dehydrogenase loading control (Figure 8A). Thus, *ldh* mRNA may be an endogenous substrate of RNase J2. When the same experiment was repeated using an RNase J2 transcriptional depletion approach, it took 120–180 min. before any reduction in RNase J2 abundance was apparent, while the first signs of aggregation in liquid culture only appeared 180 min. after inducer washout (Figures 8B,D). Overall, the results indicated that the N-terminal proteolysis approach exhibits far superior performance compared to transcriptional depletion and is similarly applicable for the study of highly toxic mutations.

## Discusssion

In the current study, we report the development of the first controllable, targeted prokaryotic N-terminal proteolysis system. Importantly, our approach should be easily adaptable for use in numerous other species, as we describe a straightforward approach to identify potential sources of species-specific degrons. Other than the SsrA C-degron from the widely conserved tmRNA, we are unaware of any previous studies describing analogous sources of easily identifiable N- or C-degrons. This is likely the reason that only a few targeted proteolysis systems have been described in the literature and all exploit the SsrA C-degron (McGinness et al., 2006; Griffith and Grossman, 2008; Davis et al., 2011; Kim et al., 2011; Wei et al., 2011; Cameron and Collins, 2014). Here, we demonstrate that members of the LexA-like family of proteins are likely to be rich sources of such sequences. Since both N- and C-degrons are found adjacent to the autocleavage sites of these proteins, one can identify candidate degrons simply by locating their autocleavage sites using multiple sequence alignments (Figure 1B). LexA-like proteins can therefore serve as species-specific sources of both N- and C-degrons. Furthermore, we have shown that inverting a C-degron can transform it into a novel N-degron. Thus, a single LexA-like protein can be a potential source of two N-degrons. Currently, it is unclear whether functional C-flip degrons are typically derivable from the C-degrons of LexA-like proteins, but it seems unlikely that this would be a unique feature of the HdiR C-degron of *S. mutans*. It is also worth noting that autocleaving LexA-like proteins form a large protein superfamily containing a broad diversity of proteins in addition to LexA orthologs, including HdiR and other LexA-like regulators, bacteriophage CI repressors, and UmuD orthologs (Kelley, 2006; Butala et al., 2009). Therefore, most, if not all, bacteria should encode at least one LexA-like protein that could be examined for potential degrons. In addition, our system employs the NEDD8 UBL protein and the NEDP1 endopeptidase from the plant species *Brachypodium distachyon*. A previous study found bdNEDP1 to be highly tolerant of a broad range of reaction conditions (Frey and Gorlich, 2014) and indeed it was the only enzyme of the 3 tested to function in *S. mutans* (Figures 6A–D). One major benefit of the NEDP1 endopeptidase is its complex substrate requirements, which confer highly stringent target specificity. For example, NEDD8 is 57% identical to ubiquitin, yet NEDP1 exhibits a 60,000-fold preference for NEDD8 compared to ubiquitin and exhibits no detectable cleavage activity for the related UBL protein SUMO (Shen et al., 2005). This exceptional specificity is highly desirable to minimize off-target cleavages that may otherwise create unwanted artifacts following heterologous expression of the protease. This specificity does come at a cost, however. The bdNEDD8 tag is relatively large (75 amino acids), which could be problematic for some protein fusions. For such cases, it may be possible to resolve issues related to steric hindrance simply by inserting a flexible glycine-serine linker after the bdNEDD8-degron tag. It is worth noting that none of the protein fusions created in this study required a linker, as the fusions did not result in any deleterious impacts upon protein function. Given the large diversity of proteins that utilize NEDDylation as a posttranslational modification (Kerscher et al., 2006), NEDD8 fusions are presumably tolerated by many proteins. In fact, bdNEDD8 fusions were found to actually improve both the expression and solubility of proteins heterologously expressed in *E. coli* (Frey and Gorlich, 2014). Therefore, bdNEDD8 is likely to be broadly useful for many protein fusions. Alternative protease/tag pairs that utilize much smaller recognition epitopes, such as that of the Tobacco Etch Virus protease, could theoretically substitute for bdNEDD8/bdNEDP1, but these will likely exhibit much lower cleavage specificity as well.

Overall, we observed strong correlations between bdNEDP1 induction times and the protein abundance of GFP, luciferase, and RNase J2 (Figures 6–8). All of these proteins were produced constitutively and were mostly depleted 60–90 min. post-induction, which was considerably more efficient than the traditional transcriptional depletion approach. If even faster degradation were required, one could presumably further increase the depletion kinetics by incorporating an additional level of exogenous genetic control to either reduce target transcript abundance or its translation. While the HdiR N- and C-flip degrons were both highly efficient at mediating target protein degradation, we observed slightly more efficient activity from the C-flip degron (Figures 7A,B). We speculate that this small improvement in C-flip performance is likely as a result of its interaction with the ClpP-dependent proteases, rather than FtsH (Figures 4B,D). Previous studies of AAA+ family proteases have demonstrated a much weaker unfoldase activity from FtsH compared to either ClpXP or ClpAP, which ultimately impacts the ability of FtsH to degrade some substrates (Herman et al., 2003; Koodathingal et al., 2009). Interestingly, while the HdiR N-degron primarily targets FtsH, it also exhibits a weak specificity for the ClpP-dependent proteases (Figures 4A,C). The promiscuity of the HdiR N-degron may explain why its performance approached that of the C-flip degron, as its targeting of FtsH would be predicted to result in much slower degradation kinetics, especially for highly stable proteins such as GFP (Herman et al., 2003; Koodathingal et al., 2009). Alternatively, proteolysis by FtsH in *S. mutans* and perhaps other species may be much more efficient than has been reported for *E. coli*. In addition, we cannot exclude the potential role of unrecognized adaptor proteins that may have further influenced the efficiencies of the HdiR degrons. The HdiR N-degron is also the first reported FtsH-dependent degron in the genus *Streptococcus*. In fact, even for the model organism *B. subtilis*, we are aware of only one report of an FtsH-dependent degron and this is a C-degron with modest activity (Le and Schumann, 2009). Given the limited mechanistic understanding of substrate recognition and degradation by FtsH, the HdiR N-degron could serve as a useful tool to target model substrates for FtsH-dependent degradation.

## Author contributions

Experiments and data analysis were performed by NL, MC, and ZX. The manuscript was written by NL, JK, and JM.

### Conflict of interest statement

The authors declare that the research was conducted in the absence of any commercial or financial relationships that could be construed as a potential conflict of interest. The reviewer MG and handling Editor declared their shared affiliation.
