# Supplementary material for: Identification of New Degrons in Streptococcus mutans Reveals a Novel Strategy for Engineering Targeted, Controllable Proteolysis
Source: Front Microbiol. 2017 Dec 19;8:2572. doi: 10.3389/fmicb.2017.02572 (PMC5742171; doi:10.3389/fmicb.2017.02572)
Supplement: Supplementary file 1 [file DataSheet1.PDF]

Figure S1.

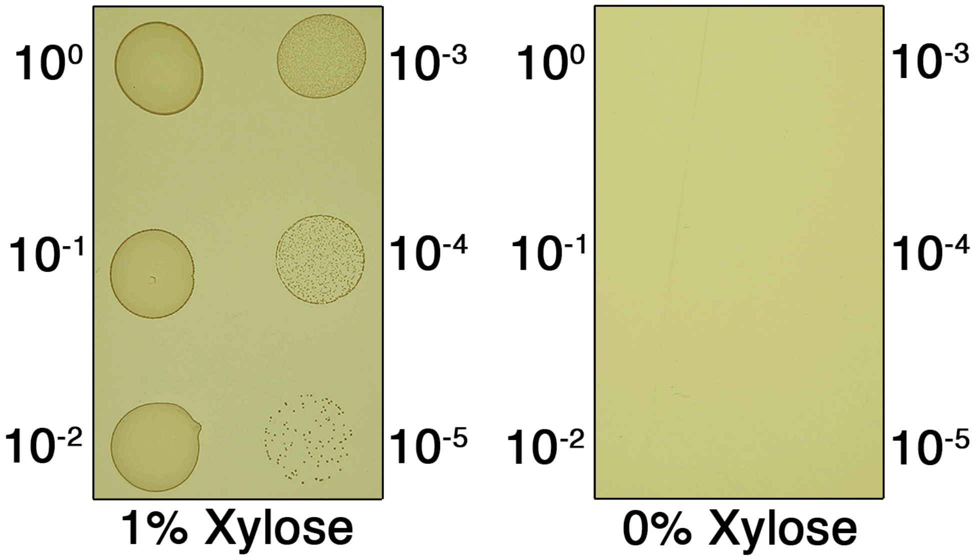

Fig. S1. FtsH is an essential protease. The *ftsH* ORF was transcriptionally fused to the Xyl-S1 induction cassette (Xie et al., 2013) to create a conditional lethal mutant strain. The mutant strain was cultured in the presence of xylose inducer and then spotted onto agar plates ± xylose inducer.

Figure S2.

Codon-optimized bdNEDP1

```
ATGGATGAAC GTGTTTTATC TTATGGTGAT GTTGTTTTAT TACGTTCTGA TTTAGCTATT
CTTCGTGGTC CTCATTTTTT AAATGATCGT ATTATTGCTT TTTATTTAGC TCATTTATCT
GCTTCTTTTC ATGGTGATGG TGATTTACTT TTATTACCTC CTAGTATTCC TTATTTACTT
TCTAATTTAC CTGATCCTGA ATCTGTTGCT GAACCTTTAT GTTTAGCTTC TCGTCGTTTA
CTTTTATTAC CTGTTAATGA TAATCCTGAT GCTTCTGTTG CTAATGGTGG TTCTCATTGG
ACTTTATTAG TTCTTGATGC TGCTACTACA GATCCTCAAG CTCCTGGTTT TGTTCATCAT
GATTCTTTAC GTGGTTCTGC TAATGCTGCA GCTGCACGTC GTTTAGCTCG TGCTTTAACT
GCTGGTGGTG CTCCTTTACG TTTTGTTGAA GCTCCTACTC CTACTCAACG TAATGGTCAT
GATTGTGGTG TTTATGTTTT AGCTGTTGCT CGTGCTATTT GTGGTTGGTG GGGTTCTTCT
CGTCGTCGTG AAAATCAACA AGGTGGTGGT GGTGATTGGT TTGCTACTAT GATGGAAGAA
GTTGATGCTG AATCTGTTGG TGCTATGCGT GCTGAATTAC TTCAATTAAT TCATCGTTTA
ATTCAAGATA AAGAACAAGA AGAAGAAAAA AAATCTAAAG CTGGTGTTGA AGATACATGT
GGTCAATAA
```

Codon-optimized bdNEDD8

```
ATGACCATGATTAAGGTGAAGACGTTGACGGGCAAGGAAATCGAGATTGATATCGAACCTACCGATACCATTGACCGTA
TCAAAGAGCGCGTCGAAGAAAAAGAAGGCATCCCGCCCGTCCAGCAACGCTTAATCTATGCCGGTAAGCAATTAGCCGA
TGATAAGACGGCTAAGGACTATAACATTGAAGGCGGATCAGTTTTACACTTAGTGTTAGCCTTGAGAGGTGGC
```

Fig. S2. Sequences of codon-optimized bdNEDP1 and bdNEDD8. Both bdNEDP1 and bdNEDD8 were synthesized after codon-optimizing their sequences for expression in *Streptococcus mutans*.

REFERENCES:

Xie, Z., Qi, F., and Merritt, J. (2013). Development of a tunable wide-range gene induction system useful for the study of streptococcal toxin-antitoxin systems. *Appl Environ Microbiol* 79**,** 6375-6384.
